# Simulating Transport Across the Blood–Brain Barrier Endothelial Membrane: Comparing Unbiased Molecular Dynamics Methods to Enhanced Sampling Protocols

**DOI:** 10.3390/membranes16100343

**Published:** 2026-10-16

**Authors:** Jerry Oluwasegun, Ben William Franey, Christian Jorgensen

**Affiliations:** 1 School of Medicine, Pharmacy and Biomedical Sciences, Faculty of Science & Health, University of Portsmouth, Portsmouth PO1 2DT, UK; up2305551@myport.ac.uk (J.O.); ben.franey@port.ac.uk (B.W.F.); 2 Institute for Life Sciences and Health, University of Portsmouth, Portsmouth PO1 2DT, UK

**Keywords:** molecular dynamics simulations, blood–brain barrier, permeability, efflux, in vitro assays

## Abstract

The blood–brain barrier (BBB) is a highly selective membrane that restricts the delivery of therapeutics into the brain. In this review, we consider a number of computational techniques that can be used to assess BBB transport and central nervous system (CNS) delivery. We outline the details of human brain microvascular endothelial cells (hBMECs) and how passive transport is the main mode of transport. We then outline details of molecular dynamics techniques for predicting in silico drug permeability across an atomistic hBMEC model. Due to the very slow timescales required to sample CNS transport a priori, we compare transition-based counting (TBC) methodology to enhanced sampling techniques. These advanced methods overcome traditional sampling limitations and include umbrella sampling (US) with the Weighted Histogram Analysis Method (WHAM) and the Dynamic Histogram Analysis Method (DHAM). Additionally, we consider constant velocity-steered molecular dynamics (cv-SMD)/US combined with the Inhomogeneous Solubility–Diffusion (ISD) model, as well as Metadynamics and the Adaptive Biasing Force.

## 1. Introduction

Neurological conditions include Alzheimer’s, Parkinson’s, and epilepsy, among others. It has been estimated that 1 in 3 adults are affected by at least one neurological condition [1], with ~3 billion people worldwide living with a neurological condition [2]. These neurological conditions affecting humans are directly or indirectly tied to the central nervous system (CNS) and its unique anatomical features. One of the important peculiarities of the brain and spinal cord is that they are separated from the blood by highly selective permeability barriers: the blood–brain barrier (BBB), the blood–spinal cord barrier, and the blood–cerebrospinal fluid barrier (BCSFB) in the choroid plexus [3]. The discovery of the BBB by Paul Ehrlich in the 1800s was a pivotal point in neuroscience history. He observed a pattern that when vital dyes are administered into the bloodstream, they stain all organs except the brain and spinal cord, which he concluded was due to a lower affinity for the dyes in the CNS than in other tissues [4].

The BBB is one of the specialised cell structures in the brain, formed by tight junctions, minimal transcytotic vesicles, and human brain microvascular endothelial cells (hBMECs) with a high density of mitochondria to support active transport, forming a close-knit assembly known as the neurovascular unit [5]. The hBMECs [6] form the primary physical barrier that regulates passive diffusion and active transport of drugs across the BBB. They are characterised by complex BBB membranes with tightly sealed intercellular junctions [7] and have a unique lipidomic signature. These complex membranes of the BBB have evolved to fulfil specific functional requirements [8] and are involved in ongoing remodelling for homeostasis, cell signalling, transport, and morphogenesis, while still acting as a cell-environment interface, providing a high degree of plasticity and stability [9]. The lateral organisation of the BBB complex membranes is characterised by its heterogeneity and chemical diversity, which are sometimes influenced by their structure and environment, contributing to why these membranes remain poorly understood [10]. These characteristics help maintain the integrity of the surrounding tissues and cells [11,12]. Key features include: selective permeability (passing certain molecules and ions while restricting others, which is essential for maintaining the cell’s internal environment) [13], phospholipid bilayer structure (two layers of phospholipids, with hydrophilic heads and hydrophobic tails) [14], protein integration (integral membrane proteins and peripheral proteins that perform specific functions in transport, signalling, and enzymatic activities) [15], polarity (distinct apical and basolateral regions with different compositions and functions) [16].

In the modern drug discovery pipeline, the ability to accurately predict the translocation and permeability coefficients of compounds from the classic benchmarks like ethanol and diazepam to novel Food and Drug Administration (FDA)-approved therapies such as esketamine [17,18] and zuranolone [19,20] is important in reducing poor pharmacokinetics and other clinical attrition. The BBB remains the primary physiological bottleneck in the development of more neuropharmacological agents, with only about 2–6% of small-molecule drugs succeeding in achieving therapeutic concentrations in the CNS [21].

Several in silico studies have attempted to simulate molecular transport across the BBB membrane; however, the computational demands and time scales required to accurately sample molecule transport across complex membranes tend to be long and demanding [22]. Traditional empirical methods and simple lipid bilayers [23] often fail to accurately capture the complex free energy landscapes explored and sampled by drug molecules during rare events while crossing the heterogeneous hBMEC lipid bilayer [22]. The use of all-atom molecular dynamics (MD) simulations to study biomolecular systems, from small molecules (sometimes anaesthetics or peptides) to large protein complexes, has improved rapidly over the past few decades [22,24], and employing enhanced sampling techniques has become the time-saving gold standard for providing atomic-level insights into the thermodynamics and kinetics of membrane crossing [25]. In this review, we will introduce a set of unbiased and enhanced sampling MD simulation methodologies and make comparisons of their pros and cons within the context of small-molecule permeation across the BBB endothelium. We will use the metrics of experimental permeability (*P*_app_) and simulated permeability (*P*_sim_) as benchmarks to ensure translational comparison, where possible.

In both experimental and computational models, it is important to acknowledge the limitations. Due to computational hardware constraints and the slow transmembrane permeability across the endothelial membrane, it is currently not routine to include all membrane components, such as the presence of all native membrane proteins, when sampling permeation. Similarly, in experimental work, the human colorectal adenocarcinoma cell line (Caco-2) is routinely used as a proxy for a number of biological barriers, including the BBB [26].

## 2. The BBB Endothelium—An Example of a Complex Membrane

### 2.1. Components of the BBB: Endothelial Cells, Tight and Adherens Junctions

The endothelial cells of the BBB form tight junctions between adjacent cells at the apical (lumen) part of the cell, conjoined to the adherens junctions [27]. The presence of these tight junctions blocks the paracellular transport of small molecules, thus rendering passive transport to be the main mechanism of action. The endothelial cells secrete a thin vascular basement of 30–40 nm membrane, composed of collagen IV, which provides structural integrity, fibronectin, laminin, heparan sulphate, and other extracellular matrix proteins [28]. Between two endothelial cells at the vascular basement membrane, tight and adherens junctions are found, formed by the homophilic interactions between transmembrane cadherin proteins and intracellular catenin proteins, Nectin, JAM, Claudin, Occludin, and others [27]. These specialised cellular structures serve a crucial regulatory function in the endothelial microenvironment, controlling how solutes, membrane proteins, and large lipid molecules move through the spaces between adjacent cells [29].

### 2.2. Other Components of the BBB

Astrocytes play a significant role in ensuring neuronal metabolism, neurotransmitter and ion uptake/recycling, synapse formation, and other biological functions of the brain beyond BBB regulation [30,31]. Additionally, they guide the formation, maturation, and modification of synaptic connections, directly influencing the neural circuit development of the CNS [32]. Pericytes are highly abundant in brain capillaries, with different densities in each vascular bed [33]. They exhibit various biological functions in association with the endothelial cells and play an important role in maintaining microvascular stability, BBB integrity, and angiogenesis [34]. The basement membrane (BM) is a unique form of extracellular matrix; it provides structural support, cell anchoring, and signal transduction [35]. There are two types of BM layers in the CNS, separated by the pericytes; the first is the endothelial BM and the second is the parenchymal BM [36]. While simulation models do not currently capture all these components of the BBB, the BBB endothelial membrane serves as a reasonable proxy of the whole BBB.

### 2.3. Functions of the BBB

The BBB provides a controlled microenvironment that functions in ion regulation by maintaining the concentration of potassium (K^+^) in the brain (∼2.5–2.9 mM) below the concentration in the mammalian body (∼4.5 mM) [31]. The concentration levels of other ions, including calcium and magnesium, are also actively regulated in the BBB. The BBB contains specific transport proteins and ion-selective channels that maintain optimal conditions for nerve cell communication and function [33]. An example is the abluminal sodium pump (the Na^+^ and K^+^-ATPase), which is responsible for maintaining a high concentration of Na^+^ and low levels of K^+^ in brain interstitial fluid (ISF) via transporting Na^+^ into the brain and K^+^  out of the brain. Another example is the luminal Na^+^, K^+^, Cl^−^ cotransporter, which facilitates the transfer of Na^+^, K^+^, and 2Cl^−^ from the blood to the endothelium. Calcium transporters (the Na^+^/Ca^2+^ exchanger) and voltage-gated K^+^ channels also regulate the ion transport across the BBB [37]. Although some metabolites and essential water-soluble nutrients are required by nervous tissue, the BBB allows low passive permeability [38]. Neurotransmitters are shared across the CNS and the peripheral nervous system (PNS). The transfer of these neurotransmitters from the brain to blood is primarily dependent on the Na^+^-coupled and Na^+^-independent amino acid transporters. The BBB regulates the entry and transport of certain amino acids including the neurotransmitters glutamate and glycine, while actively transporting many essential amino acids out of the brain [7].

### 2.4. Small-Molecule Delivery Through Complex Membranes

In biological research, molecules that can easily cross cell membranes and be available in the cytosol are consistently needed. Permeable molecules like fluorescent dyes, crosslinking molecules, fluorogenic enzyme substrates, and various protein inhibitors have been useful for biological research over the years [39]. Molecules may be transported by natural or artificial delivery systems or passively diffuse across the cell membrane [10]. Significant effort has gone into the study of small-molecule permeation across membranes [40,41,42,43], but these are not the only permeants of interest. As an example, peptides [44,45,46,47,48] are emerging as important therapeutics with oncological potential that rely on transmembrane delivery. Such studies have highlighted the importance of several molecular parameters in modulating the transport, including molecular weight and size, polarity, charge distribution, hydrophobic versus hydrophilic properties, and solubility. Small molecule transmembrane transport is generally determined by two distinct mechanisms: active transport and passive diffusion. However, in transport across the BBB, tight junctions between adjacent endothelial cells restrict paracellular passive diffusion, leaving transmembrane diffusion as the primary passive mechanism. Computational models of the BBB can therefore achieve reasonable accuracy when just including the endothelial membrane component, but this may not be the case for other types of complex membranes.

Most small-molecule drugs targeting the CNS enter the brain through passive diffusion across the BBB, which requires more restrictive physicochemical criteria than Lipinski’s rules of 5 suggest [49]. These rules are readily broken by a considerable fraction of the drug space (estimated at 10^60^ molecules) [22,50,51], which has given rise to the notion of non-Lipinski behaviour. Lipophilicity, measured by log*P*, appears to be statistically significant with different types of passive transport across the endothelial barrier [52,53,54,55]. While some authors have argued that generally lipophilic molecules cross the BBB endothelium [56], other analyses have shown that moderately hydrophilic or even very lipophilic molecules cross the BBB endothelium significantly faster than more lipophilic molecules [52]; such analyses focus on the permeability of hydrophilic molecules including ethanol (*P_app_* 1.1 × 10^−3^ cm s^−1^) [57] and propanol (*P_app_* 3.3 × 10^−3^ cm s^−1^) [57].

## 3. Molecular Modelling Across the Endothelial Membrane

### 3.1. Basics of MD Simulations

We proceed to outline some basic principles of molecular modelling. In silico techniques such as Molecular Dynamics (MD) simulations provide key insights when evaluating highly restrictive environments like the BBB.

**Figure 1:**
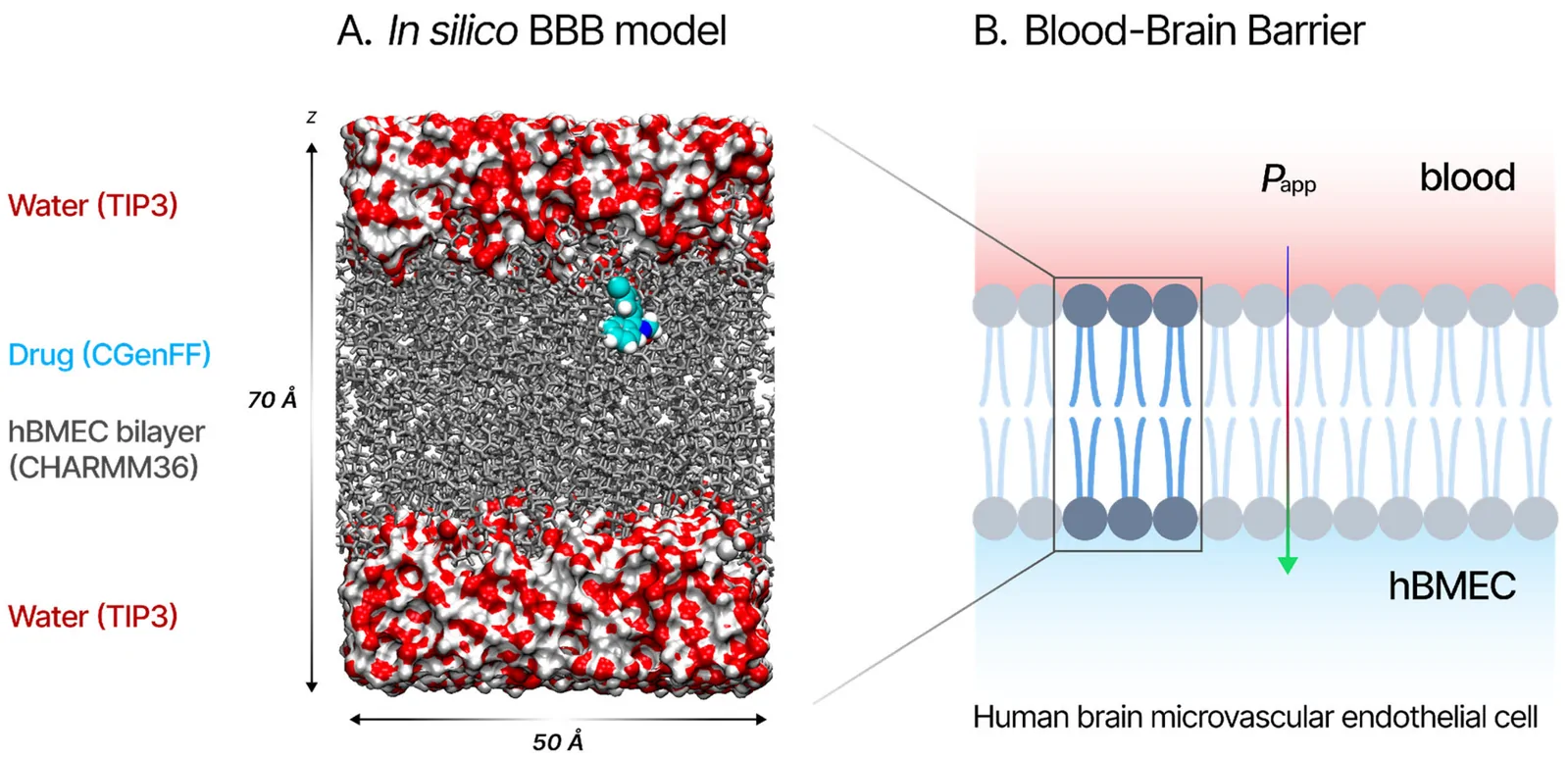
Atomistic system design of the hBMEC model. (**A**) Atomistic representation of the heterogeneous human brain microvascular endothelial cell (hBMEC) membrane model. The bilayer features a high-cholesterol (29.1%) lipidomic signature with simulation box dimensions of 50 Å × 50 Å × 70 Å, denoted by arrow dimensions, which has at least ~25 Å of bulk water on either side. (**B**) Schematics of the endothelial (hBMEC) BBB component, showcasing the direction of travel from apical to basolateral experimental permeability (P_app,A→B_) with a vertical arrow.

In MD simulations, one solves Newton’s second law equation of motion for all atoms in the system. This requires the numerical integration of the equations of motion through algorithms such as the Verlet algorithm, leap-frog algorithm, or velocity Verlet method [58,59]. MD simulations can describe complex membranes accurately thanks to the type of ensemble employed. Specific thermodynamic ensembles keep certain parameters fixed during the simulation, allowing others to vary. Simulations often start in the canonical (NVT) ensemble, with a constant number of particles, volume, and temperature, to allow the membrane-water box system (Figure 1) to equilibrate. This is typically followed by further equilibration and production runs in the isothermal-isobaric (NPT) ensemble with a constant number of particles, pressure, and temperature. The pressure in the simulation box is controlled by a barostat, with choices including the Berendsen barostat (weak coupling to external pressure bath) [60], or the Parrinello–Rahman barostat (allowing box shape changes for stress calculations) [61,62].

One of the biggest pitfalls in using MD simulations to study membrane permeation arises when the underlying energy function describing the system box does not accurately represent the biological process of permeation. This energy function, termed the force field (FF), is one of the factors that require the use of state-of-the-art models. Several specialised FFs have been developed for different types of systems, including AMBER (Assisted Model Building with Energy Refinement) primarily for proteins and nucleic acids [63], CHARMM (Chemistry at Harvard Macromolecular Mechanics) for biomolecules [64], GROMOS (Groningen Molecular Simulation) for biomolecular system [65,66], OPLS (Optimised Potentials for Liquid Simulations) [67], originally for liquid simulations but now widely used for biomolecules, OpenMM FF [68], and MMFF (Merck Molecular Force Field) [69] designed for small organic molecules. One common water model is TIP3P [70].

In Figure 1A, we depict an atomistic model of the BBB and the corresponding endothelial (hBMEC) BBB membrane it seeks to mimic (Figure 1B).

We briefly describe the exact potential energy $U\left({\underline{r}}^{N}\right)$ as a function of the coordinates $\underline{r}$ of the *N*-atom system energy for the experimentalist reader. This function is given in terms of internal coordinate bonded interactions and pairwise non-bonded interactions (Equation (1)):

(1) $$U_{\mathrm{FF}}\left({\underline{r}}^{N}\right)=U_{\mathrm{Bonded}}\left({\underline{r}}^{N}\right)+\;U_{\mathrm{Non}-\mathrm{Bonded}}\left({\underline{r}}^{N}\right)$$

The bonded interaction potential represents the intramolecular forces and is a sum of bond stretching, angular bending, torsional (dihedral) terms, Urey–Bradley cross-terms, and improper dihedrals. Specifically, the bond stretching potential is modelled harmonically as $K_{b}{\left(b-b_{0}\right)}^{2}$; the 1-2-3 angle bending potential is given by $K_{\theta }{\left(\theta -\theta_{0}\right)}^{2}$; the 4-body dihedral (torsional) term is represented by a Fourier series *K_χ_*; the Urey–Bradley term accounts for 1,3-nonbonded interactions using a harmonic potential $K_{\mathrm{UB}}{\left(S-S_{0}\right)}^{2}$; and the improper dihedral term, used to maintain specific molecular geometries (like planarity), is given by $K_{\phi }{\left(\phi -\phi_{0}\right)}^{2}$. The total bonded potential is given by Equation (2):

(2) $$\begin{array}{cc} U_{\mathrm{Bonded}}\left({\underline{r}}^{N}\right) & =U_{\mathrm{Bonds}}+U_{\mathrm{Angle}}+U_{\mathrm{Dihedral}}+U_{\mathrm{Urey}-\mathrm{Bradley}}+\;U_{\mathrm{Improper}}\\ & =\sum_{\mathrm{bonds}}{K_{b}\left(b-b_{0}\right)}^{2}+\sum_{\mathrm{angles}}{K_{\theta }\left(\theta -\theta_{0}\right)}^{2}+\sum_{\mathrm{dihedrals}}K_{x}\left(1+\cos\left(\mathrm{n\chi}-\delta \right)\right)\\ & +\sum_{\mathrm{Urey}-\mathrm{Bradley}}{K_{\mathrm{UB}}\left(S-S_{0}\right)}^{2}+\sum_{\mathrm{impropers}}{K_{\phi }\left(\phi -\phi_{0}\right)}^{2} \end{array}$$

where *K_b_*, *K_θ_*, *K_χ_*, *K_UB_* and *K_ϕ_* are the force constants of the bond stretching, angle bending, dihedral, Urey–Bradley, and improper terms, respectively. *b*, *θ*, *χ*, *S* and *ϕ* are the instantaneous bond length, valence angle, dihedral angle, 1,3 distance, and improper angle. The subscript 0 denotes the corresponding equilibrium value, *n* is the dihedral multiplicity, and *δ* is the dihedral phase shift.

The non-bonded interaction potential accounts for intermolecular forces and intramolecular forces between atoms separated by three or more bonds. It is the sum of van der Waals forces (*U_VdW_*) and electrostatic interactions (*U_el_*). The van der Waals forces are modelled using the Lennard–Jones potential, which relies on the well depth *ε_ij_* and the minimum distance *R_min_*, while the electrostatic interactions are modelled using Coulomb’s law with the atomic partial charges *q_i_* and *q_j_*. The total non-bonded potential is given by Equation (3):

(3) $$\begin{array}{cc} U_{\mathrm{Non}-\mathrm{Bonded}}\left({\underline{r}}^{N}\right) & =U_{\mathrm{VdW}}+U_{\mathrm{el}}\\ & =\sum_{\mathrm{Non}-\mathrm{Bonded}}\left(\varepsilon_{\mathrm{ij}}\left[{\left(\frac{R_{\min}}{r_{\mathrm{ij}}}\right)}^{12}-2\;{\left(\frac{R_{\min}}{r_{\mathrm{ij}}}\right)}^{6}\;\right]+\frac{q_{i}q_{j}}{{4\pi \varepsilon }_{0}r_{\mathrm{ij}}}\right) \end{array}$$

where *r_ij_* is the distance between atoms *i* and *j*, and *ε*_0_ is the vacuum permittivity.

### 3.2. Simulating Drug Delivery Through the BBB Endothelium

All-atom MD simulations have proved important in allowing for mechanistic observations not previously visible experimentally [71], including: (1) energy barriers for passive diffusion, (2) properties including lipophilicity, size, and charge, as well as (3) membrane passage thermodynamics and kinetics that can be translated to phenomena critical for drug bioavailability [72]. In protein-mediated transport, MD simulations can model drug interactions with the transporters, such as P-glycoprotein (P-gp) or ATP-Binding Cassette (ABC) transporters. For example, these simulations can identify major and minor binding modes, most important interaction residues, and conformational changes, all of which take place during the transport cycle. This can aid in the design of drugs that will evade efflux transporters or target uptake transporters [73,74]. Recent advances to tackle the issue of P-gp efflux for antibiotics and chemotherapeutics have seen scaffold redesign to improve the efflux profile of the drug [74,75].

If simulation timescales are too prohibitive with All-atom MD, coarse-grained (CG) MD simulations (CGMD), such as the MARTINI 3 FF and lipid FF [76], present a promising avenue to achieve such timescales, for example, for studying efflux of molecules out of the BBB. Early CG simulations of P-gp have already looked at the interaction of P-gp with the substrate molecule at timescales over 50 μs [77,78], while multiscale simulations have been able to explore P-gp conformational transitions and substrate access [79,80]. Similarly, a better description of the glycan coating of the BBB and associated efflux pumps like P-gp will result in more accurate models. Advances like GlycoShape [81] offer the possibility to more accurately model this.

Despite their utility, MD simulations face several limitations. For instance, time scale limitations typically restrict MD simulations to nanoseconds or microseconds, while many biological processes occur over milliseconds to seconds. Additionally, force field accuracy is constrained by inherent approximations that may not accurately represent all molecular interactions. Furthermore, computational cost becomes prohibitive for large systems or long-time scales, and classical MD cannot capture quantum mechanical effects like tunnelling or electronic transitions. Finally, achieving adequate sampling of conformational space remains challenging for complex systems. However, recent advances have addressed some of these limitations. For example, polarisable force fields now account for electronic polarisation effects in response to the local electric field [82]. Coarse-grained models reduce computational cost by representing groups of atoms as single interaction sites. Quantum Mechanics (QM)/MM hybrid methods combine quantum mechanical and molecular mechanical approaches for reactive regions. Additionally, machine learning potentials use artificial intelligence to develop more accurate interatomic potentials based on quantum mechanical calculations, and enhanced sampling techniques improve the exploration of conformational space.

### 3.3. Biophysical Principles of Passive Membrane Permeation Across the hBMECs 

The passive permeation of a solute across a lipid bilayer is fundamentally a multi-step process involving desolvation from the bulk water phase, insertion into the lipid headgroup region, translocation across the hydrophobic core, and re-solvation in the distal aqueous phase [83]. The standard theoretical framework for this process is the ISD framework, which treats the membrane as a series of resistive layers. The permeability coefficient (*P*_sim_) is defined by the integral resistance across the membrane normal (*z*), as shown in Equation (4) [22,84,85]:

(4) $$\frac{1}{P_{\mathrm{sim}}}=\int_{z_{1}}^{z_{2}}\frac{\exp\;\left(\mathrm{\beta G}\left(z\right)\right)\;}{D\left(z\right)}\mathrm{dz}$$

In Equation (4), the Potential of Mean Force (PMF) or free energy profile is denoted by *G*(*z*), and *D*(*z*) denotes the position-dependent diffusion coefficient [86]. $\beta =\frac{1}{k_{B}\;T}$, where *k_B_* is the Boltzmann constant and *T* is the absolute temperature, and *z*_1_ and *z*_2_ are the positions in the bulk water on either side of the bilayer along the membrane normal *z*. Accurate measurement of permeability coefficient requires the precise determination of both the thermodynamic barrier ΔG and the kinetic resistance (1/*D*) [87]. The complexity of the hBMEC membrane, with its high cholesterol content and dense packing, introduces significant challenges for both variables [88]. The thermodynamic resistance and free energy barriers reflect the probability of finding the drugs at a specific depth within the membrane; for hydrophobic drugs like diazepam, esketamine [89] and zuranolone, the PMF often exhibits a local minimum at the interface between the lipid headgroups and the acyl tails, followed by a variable barrier at the bilayer centre. Accurate calculation of *G*(*z*) is one of the primary objectives of both umbrella sampling and SMD methodologies, though they both achieve this through fundamentally different statistical mechanical pathways [43,90].

The diffusion coefficient *D*(*z*) within a lipid bilayer is strongly anisotropic and position-dependent. Diffusion is high and nearly isotropic in the aqueous phase but decreases by several orders of magnitude in the headgroup region due to increased local viscosity and electrostatic interactions [86]. In the hBMEC model, cholesterol further suppresses both lateral and transverse diffusion through its condensing effect on the membrane [91,92].

ISD models often rely on the Einstein-Smoluchowski relation, which estimates the permeability of a small-molecule solute from the PMF and the diffusion coefficients *D*(*z*) of the drug as a function of its position along the transmembrane axis (*z*) [40]. The ISD equation has been used in combination with simulation data to successfully estimate the experimental permeability in a number of applications and case studies [22,43,85,87,93,94,95].

During BBB small-molecule permeation, the *P*_app_ can be affected by P-gp-mediated efflux [96], in which molecules crossing into the brain that are substrates of P-gp are effluxed back out. To handle this effect experimentally, assays have progressed from the standard in vitro cell models Caco-2 and Madin-Darby Canine Kidney (MDCK) [97] to more specialised assays including the MDCK cell line transfected with Multi-Drug Resistance 1 permeability assay (MDCK-MDR1), which has proven to be one of the gold standards for spotting the potential P-gp efflux substrates [98].

It should be noted that a direct comparison of *P*_app_ and *P*_sim_ is often difficult to make without incurring several assumptions in either direction, as discussed elsewhere [22]. The first observation is that because passive transcellular transport is dominant rather than via paracellular routes (tight junction pathways), simulation models that suppress efflux effects can still be relied on, but for efflux active compounds the efflux ratio can surpass 10, meaning there is a significant differential between *P*_app,A→B_ and *P*_app,B→A_, and for which a direct comparison to *P*_sim_ is rendered more difficult [73]. Secondly, the models should have reasonably matching biophysical conditions. The simulation lipid composition should reasonably mirror the target membrane’s key physical properties, including phase behaviour, fluid state, acyl chain saturation, and major headgroup classes.

Other studies have also shown that the overlooked hydrodynamic barrier of the unstirred water layer (UWL) in the actual experiments creates a zone where the diffusional movement of molecules can exceed convection, which reduces the *P_app_* of fast-permeating compounds [99] and is not represented in *P_sim_*, contributing to the orders-of-magnitude differences between simulated and experimental permeability values [100].

### 3.4. Atomistic Models of the BBB

The physiological relevance of in silico permeability studies is strongly dependent on the accuracy of the model membrane composition. The hBMEC apical membrane differs substantially from generic bilayers or porcine brain lipid extracts due to its elevated sphingomyelin and cholesterol content [101], which promotes the formation of a restrictive liquid-ordered phase compared to the liquid-disordered phase of simple phosphatidylcholine bilayers [23]. An example is the membrane composition developed by Jorgensen et al., derived from experimental lipidomics data of human brain microvascular endothelial cells [91] (Table 1). BBB simulation models can be built with a variety of tools, including the CHARMM-GUI Membrane Builder [102] utilising the CHARMM36 lipid force field [103] and the TIP3P water model [70].

### 3.5. Other Atomistic Bilayers Beyond the BBB Endothelium

**Figure 2:**
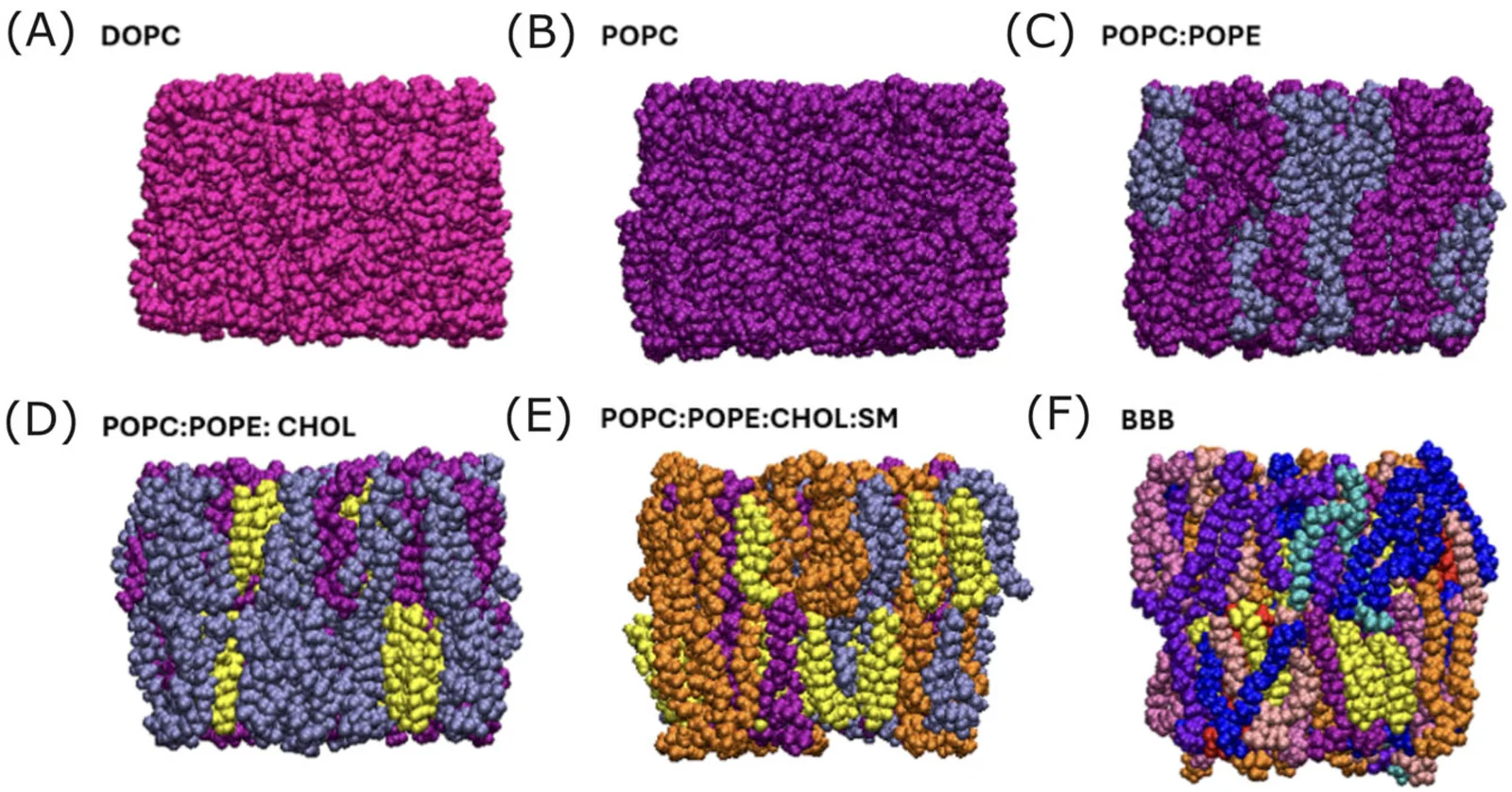
Lipid membrane models of increasing compositional complexity. (**A**) DOPC, (**B**) POPC, (**C**) POPC:POPE with a 70%:30% composition, (**D**) POPC:POPE:CHOL with a 35%:35%:30% composition, (**E**) POPC:POPE:CHOL:SM with a 20%:20%:30%:30% composition (SM is represented by OSM for the CHARMM-GUI construction), and (**F**) the blood–brain barrier (BBB) model containing POPC, SAPC, SLPC, SAPE, SOPE, CHOL, OSM, SAPS, and SAPI. All lipid compositions and ratios were selected from previously reported membrane models. All membranes are shown with 96 lipids for visual consistency. Phosphatidylcholines (PC) are shown in shades of purple/pink (POPC in purple, SAPC in mauve, SLPC in violet2, and DOPC in magenta2). Phosphatidylethanolamines (PE) are shown in shades of blue (SAPE in blue, SOPE in cyan, and POPE in ice blue). Cholesterol (CHOL) is shown in yellow, OSM in orange, SAPI in red, and SAPS in pink. All lipids are rendered in Van der Waals representation. Lipid membranes were generated using CHARMM-GUI [102] and visualised in VMD.

Unlike simple, liquid-disordered phosphatidylcholine bilayers, the apical membrane of hBMECs contains elevated concentrations of cholesterol and sphingomyelin [23]. These sterol-sphingolipid interactions drive the formation of a highly restrictive liquid-ordered phase, which significantly decreases the area per lipid, reduces lateral compressibility, and increases lipid order parameters within the hydrophobic core [91,92]. To accurately capture the thermodynamic barriers to passive diffusion, computational models must progress beyond simplified setups. Figure 2 illustrates this comparative progression from simple single- and binary-component bilayers to a highly heterogeneous, 96-lipid BBB model. This 96-lipid assembly was derived from experimental lipidomics data to serve as a computationally efficient yet biophysically relevant representation of the hBMEC membrane [91].

## 4. Enhanced Sampling Methods

Several enhanced sampling techniques have been developed with differing methodologies, advantages, and limitations for the study of membrane permeation. The utility of these methods depends on their validation against experimental benchmarks, therefore requiring assessment of computational convergence, collective variable (CV) selection, and statistical precision to accurately predict *P*_sim_ relative to experimentally determined in vitro *P*_app_ values for CNS therapeutics [22]. Among other enhanced sampling techniques [24,83], this review explores the use of equilibrium-based umbrella sampling (US) analysed with the Weighted Histogram Analysis Method (WHAM), Dynamic Histogram Analysis Method (DHAM) [86,104], and constant velocity (cv)-steered MD (cv-SMD)/US with Inhomogeneous Solubility–Diffusion (ISD) model [105] to accurately measure the simulated permeability (*P_sim_*) in comparison to the in vitro apparent permeability *P_app_*.

### 4.1. Unbiased MD Simulations with Transition-Based Counting (TBC)

The first method is unbiased MD with TBC. Here, the rate constant, *k*, for translocation across the bilayer is calculated from unbiased MD simulations of spontaneous trans-bilayer solute crossings. To do this, the total number of transport events observed during a simulation is first estimated:

*N* = *N_in_* + *N_out_* (5)

**Figure 3:**
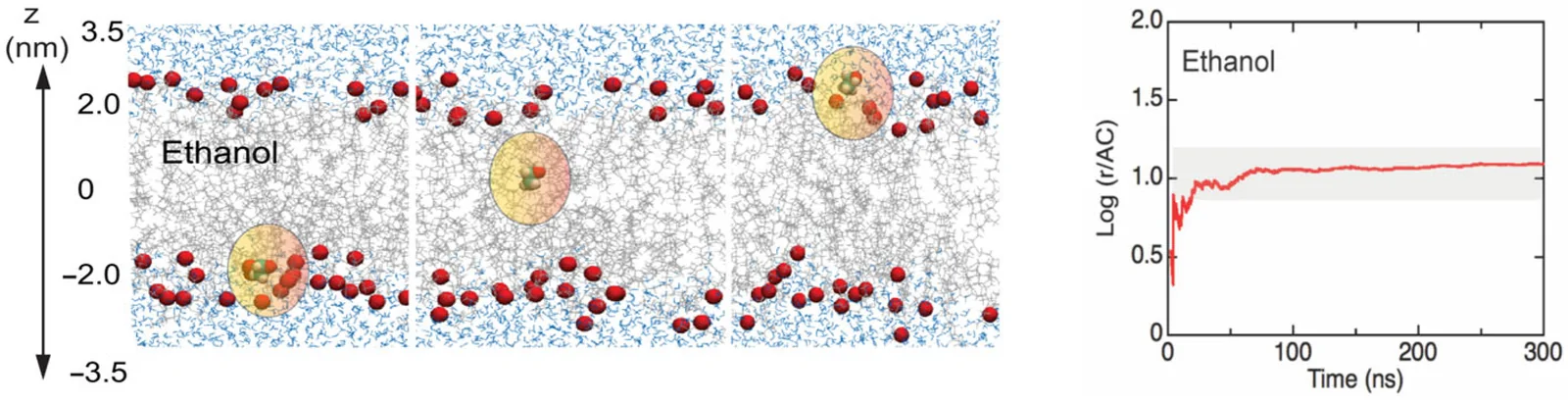
Basics of transition-based counting approaches for permeability. Transitions illustrated for the case of ethanol permeating across a complex membrane in an unbiased MD simulation, and for which we assume that passive diffusion is the primary mechanism of transport. Molar rate constant for ethanol throughout the MD simulation, revealing the time needed to converge rate estimates as an input to the permeability calculation. Red spheres indicate boundary of membrane. Blue lines illustrate the solvent (water). The orange circle highlights the molecule transitioning. Reproduced from https://doi.org/10.1021/acs.jcim.4c01815 [22]. Copyright 2025 ACS. Accessed on 1 August 2026.

This number *N* is then divided by the total simulation time:

(6) $$k=\frac{N}{t}$$

where *N_in_* and *N_out_* are the numbers of transitions across the bilayer in the inward and outward directions, respectively, and *t* is the total simulation time. It is common to use a molar rate constant (*r* = *k*/*N_A_*, where *N_A_* is the Avogadro constant) in calculations [22,40]. In Figure 3, we demonstrate this procedure:

### 4.2. Steered MD

US and Steered MD were developed to address a fundamental question in molecular biophysics: how individual protein subdomains respond to mechanical stress [90].

In one of its first applications, SMD was used to investigate the unfolding of Titin immunoglobulin domains [106]. It mimics single-molecule manipulation experiments, such as atomic force microscopy (AFM) [107], by applying an external non-equilibrium force to selected groups of atoms [90].

**Figure 4:**
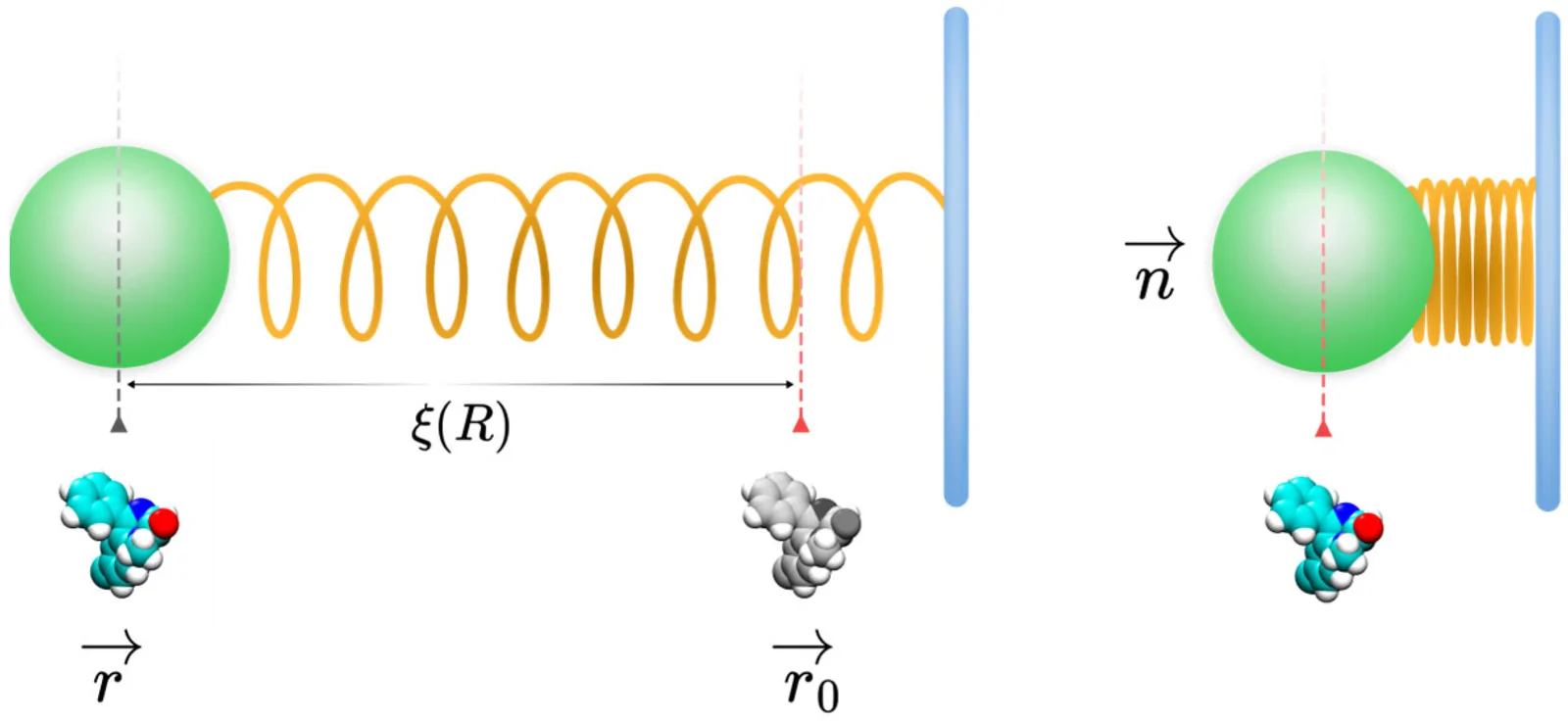
Schematic illustrating the steering direction and reaction coordinate *ξ*(*R*) of Constant Velocity Steered Molecular Dynamics (cv-SMD). The dot product $\left(\underline{r}-{\underline{r}}_{0}\right)\cdot \underline{n}$) calculates the projection of the displacement vector in the steered direction $\underline{n}$. Here, $\underline{n}$ is the direction of the steering force, *vt* (velocity × time) gives the position of the atoms along $\underline{n}$, −∇ is the negative gradient of the potential energy, and *κ* is the pulling constant as shown in Equation (7).

SMD simulations use either constant force (cf-SMD) or constant velocity (cv-SMD) to study conformational changes on timescales inaccessible to conventional MD simulations [108]. In cv-SMD, a designated atom or group of atoms (referred to as SMD atoms) is pulled toward an external reference point $\underline{r}$ at a constant velocity $\underline{v}$ using a virtual harmonic spring, where the steering force is the negative derivative of the biasing potential with respect to the collective variables/reaction coordinates *ξ*(*R*). As an example, one might employ cv-SMD to rapidly generate initial states for subsequent umbrella sampling production runs to obtain sufficient sampling for accurate reconstruction of free energy profiles. The dynamics of the spring in cv-SMD is depicted in Figure 4, and the underlying pulling equation is given in Equation (7):

(7) $$\underline{F}=-\;\nabla \left(\frac{1}{2}\kappa {\left[\underline{v}t\;-\;\left(\underline{r}-{\underline{r}}_{0}\right)\cdot \underline{n}\right]}^{2}\right)$$

where *F* is the steering force acting on the SMD atoms, *κ* is the pulling (spring) constant, *v* is the constant pulling velocity, *t* is the simulation time, $\underline{r}$ is the position of the SMD atoms, ${\underline{r}}_{0}$ is their initial position, and $\underline{n}$ is the unit vector along the pulling direction.

### 4.3. Umbrella Sampling

**Figure 5:**
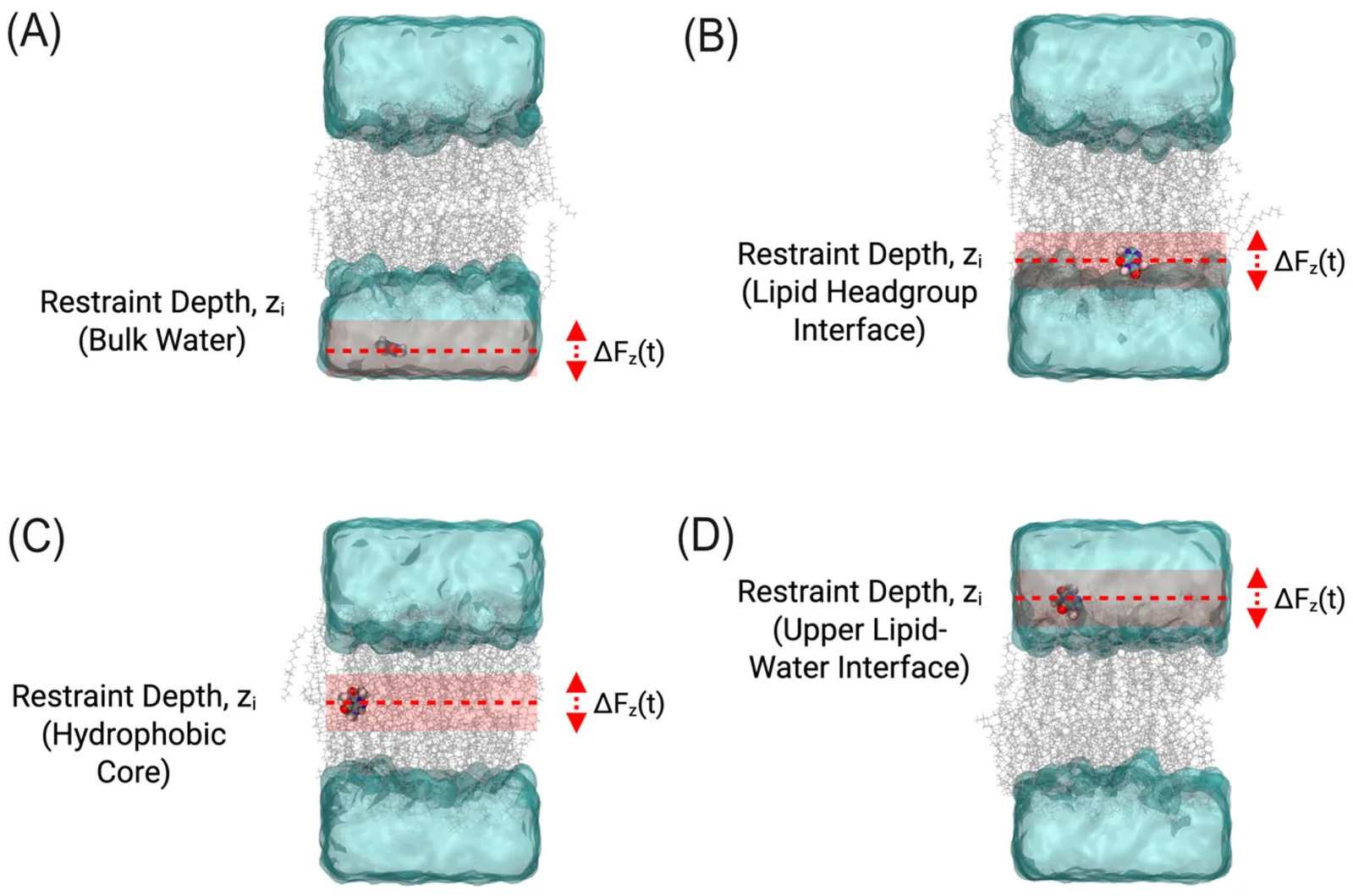
Representative snapshots of the umbrella sampling methodology across the hBMEC membrane. Sequential simulation windows showing the drug artificially restrained at specific spatial depths along the permeation pathway. The horizontal dashed lines indicate the specific restraint depth (*z_i_*) where a harmonic biasing potential (*U_bias_*) is applied to sample the local free energy landscape, including (**A**) the bulk water, (**B**) the lipid–water interface, (**C**) the hydrophobic core, and (**D**) the second lipid–water interface. The transparent red bands illustrate the positional variance allowed by the harmonic spring. The resulting force fluctuations Δ*F_z_*(*t*) (denoted by red arrows), are extracted to calculate the position-dependent diffusion coefficient *D*(*z*) via the Green–Kubo relations. The colored lines indites the restraint plane.

Umbrella sampling [109] can be used following the generation of initial windows with other methods, either via script-based generation or from applications like cv-SMD [110]. In each window, a harmonic biasing potential is applied to restrain the molecule at a specific depth *z_i_* and the windows must overlap to sample the complete reaction coordinate [111] (Equation (8)). As illustrated in Figure 5, this harmonic biasing potential restrains the drug molecule at specific depths (*z_i_*) across the membrane system, including different local environments such as the bulk water or hydrophobic core, therefore allowing for the extraction of localised thermodynamic and kinetic data.

(8) $$U_{\mathrm{bias}}\left(z\right)=\frac{1}{2}k_{i}{\left(z-z_{i}\right)}^{2}$$

**Figure 6:**
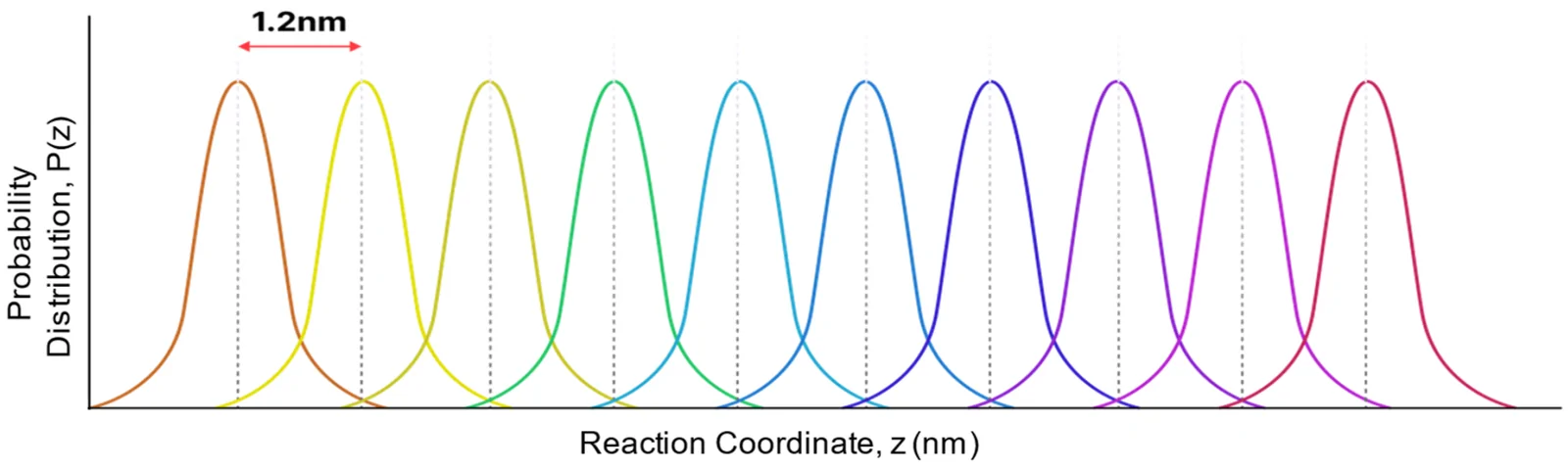
Schematic representation of the umbrella sampling windows. Representative overlapping windows are separated by 1.2 nm intervals. These intervals are used to restrain the molecule at specific depths (*z_i_*) along the permeation coordinate, allowing for complete sampling of the free energy landscape over time and ensuring the full reaction coordinate is covered.

A representative distribution of windows is given in Figure 6. The PMF is typically obtained by unbiasing the probability distributions from the restrained simulations, using the WHAM or DHAM [104,112].

Once the complete reaction coordinate is fully sampled, the thermodynamics must be reconstructed. The Dynamic Histogram Analysis Method (DHAM), designed by Rosta and Hummer [104], is a promising development that seeks to address convergence-related errors in free energy estimates obtained with WHAM. While WHAM assumes global equilibrium along the reaction coordinate, DHAM adopts a transition-based framework in which the free energy landscape is partitioned into bins and transitions between neighbouring states are quantified through a Markov transition matrix *M_ij_* [113]. By explicitly accounting for the inter-bin transitions, it reduces artifacts arising from incomplete sampling due to transient trapping. The free energy *G*(*z*) profile is recovered from the stationary distribution (*π*) of the Markov model’s dominant eigenvector, yielding a more reliable estimate of the underlying free energy surface under nonequilibrium or partially converged conditions [104] (Equation (9)).

*G*(*z*) = −*k_B_T ln*(*π*(*z*)) (9)

where *π*(*z*) is the stationary probability of finding the drug in the bin at position *z*, *k_B_* is the Boltzmann constant and *T* is the absolute temperature.

Unlike other MSM approaches, DHAM directly explores the observed transitions between spatial bins within a defined time window, enabling the calculation of rate constants from biased simulations [114]. Permeability is then determined by reconstructing the unbiased free-energy profiles and transition rates using DHAM. From the biased trajectories, the residence time in each spatial bin and the number of transitions between neighbouring bins were computed and used to fit a continuous-time master equation that satisfies detailed balance [43]. This procedure yields an unbiased PMF and position-dependent transition rates along the permeation coordinate. It should be noted that, because WHAM requires adequate equilibrium sampling within umbrella windows and sufficient overlap, the improvements that DHAM offers cannot generally correct fundamentally insufficient sampling or unresolved slow degrees of freedom, so users are still encouraged to generate good sampling in their simulation studies.

### 4.4. Umbrella Sampling-DHAM, Using Markov State Modelling

The determination of the PMF, which represents the free energy profile of a molecule as it moves through a membrane [115], was achieved through a multi-step process involving biased simulations and a specific unbiasing mathematical framework. The system was biased using umbrella sampling to ensure the drug molecule adequately sampled all regions of the membrane with especially high-energy barriers. *N* umbrella sampling windows are generated for each drug, and each window is separated by a suitable distance allowing for window overlap. The construction of MSMs and the raw transition counts from biased simulation cannot represent the true natural behaviour of the system [116]. DHAM was applied to recover the unbiased transition matrix *M_ij_*, and a maximum likelihood estimate was used to calculate the unbiased transition probabilities based on the biased counts, a known biasing energies applied during the simulation [43], can be calculated using Equation (10):

(10) $$\;M_{\mathrm{ji}}\left(t\right)=\frac{\sum_{k}C_{\mathrm{ji}}^{k\left(t\right)}}{\sum_{k}n_{i}^{k}\exp\;\left(-\frac{u_{j}^{k}-\;u_{i}^{k}}{2k_{B}T}\right)\;}$$

Here, *M_ji_*(*t*) is the unbiased probability of a transition from state *i* to state *j* at lag time *t*, $C_{\mathrm{ji}}^{k}(t)$ is the count of transitions from state *i* to *j*, in simulation *k*, $n_{i}^{k}$ is the total number of counts in state *i* in simulation *k*, and $u_{i}^{k}$ is the biasing energy applied to state *i* in that simulation.

As an example, the *Z*-coordinate can be discretised into 1000 small bins, and for each simulation the number of observed transitions was counted between these bins (*i* to *j*) at a specific lag time (e.g., 200 ps was chosen for the final analysis) [85,117]. The free energy profile can then be calculated from the equilibrium populations of the system’s states (the bins across the reaction coordinate), which were derived from the largest eigenvalue (*λ*_1_ = 1) of the transition matrix in the constructed MSM [43]. Since the simulations are biased (using harmonic potentials to ensure the drug samples all regions of the membrane), DHAM is used to unbias the data.

### 4.5. Metadynamics

Metadynamics addresses the limitations of rigid reaction coordinates by saving history-dependent bias potentials along selected CVs to discourage the system from revisiting sampled conformational states [118]. By systematically depositing history-dependent Gaussian potentials, this technique forces the drug molecule to escape deep local minima and cross high-energy barriers [118]. Variants such as Well-Tempered (WT-MetaD) and Multiple-Walker Metadynamics (MW-MetaD) can further enhance efficiency. MW-MetaD accelerates convergence by parallelising the exploration of the free-energy surface across multiple replicas that share a common bias history; WT-MetaD decreases the height of the deposited Gaussian biases over time to prevent the over-filling of free-energy basins [119]. Coupling this approach with parallel tempering (PT-MetaD) can displace systems from the restrictive liquid-ordered phases of complex membranes by exchanging configurations across temperature variations [120]. The hBMEC membrane contains cholesterol and sphingomyelin, which contribute to the formation of a restrictive liquid-ordered phase [52,92]. Drug molecules can become trapped in local minima within this heterogeneous environment; applying forms of metadynamics to the hBMEC model could displace these trapped molecules and resolve the slow orthogonal relaxation times [94,118]. However, applying history-dependent biases in these environments risks over-filling the free energy basins and disrupting the lipid structure unless the biases are carefully defined [119].

### 4.6. Adaptive Biasing Force (ABF)

Adaptive Biasing Force (ABF) continuously computes the local mean force and applies an equal and opposite biasing force to dynamically flatten the free energy landscape, meaning diffusivity can be explored across the system without predefined windows [94,121]. ABF directly measures and cancels the thermodynamic force along the permeation axis, allowing for the extraction of the free energy profiles without relying on post-simulation unbiasing frameworks [121]. When validated against experimental metrics, ABF frequently achieves statistical convergence comparable to or superior to US, provided orthogonal degrees of freedom are adequately captured [121]. However, ensuring that orthogonal relaxation times do not exceed the simulation timescale remains key in preventing quasi-non-ergodicity and hysteresis [94,121]. Applying ABF to the hBMEC membrane offers a theoretical pathway to map the free energy landscape without generating predefined spatial windows [121]. As the liquid-ordered phase is governed by slow and coupled degrees of freedom, achieving convergence with ABF would likely still require extended simulation times to ensure local lipid relaxations do not induce hysteresis [94].

### 4.7. Kinetics and Compound Translocation and Permeability Estimation

To calculate the permeability with the ISD equation, one needs firstly the PMF obtained with one of the previously mentioned methods. Secondly, one needs to estimate the position-dependent diffusion coefficient *D*(*Z*), which explains how quickly z-position fluctuations lose memory over time in each window, and is given by [122]

*D*(*Z*) = *k_B_T*/*Kτ_c_* (11)

where *k_B_* is the Boltzmann constant, *T* is the absolute temperature, and *K* is the force constant of the harmonic restraint in the window.

**Figure 7:**
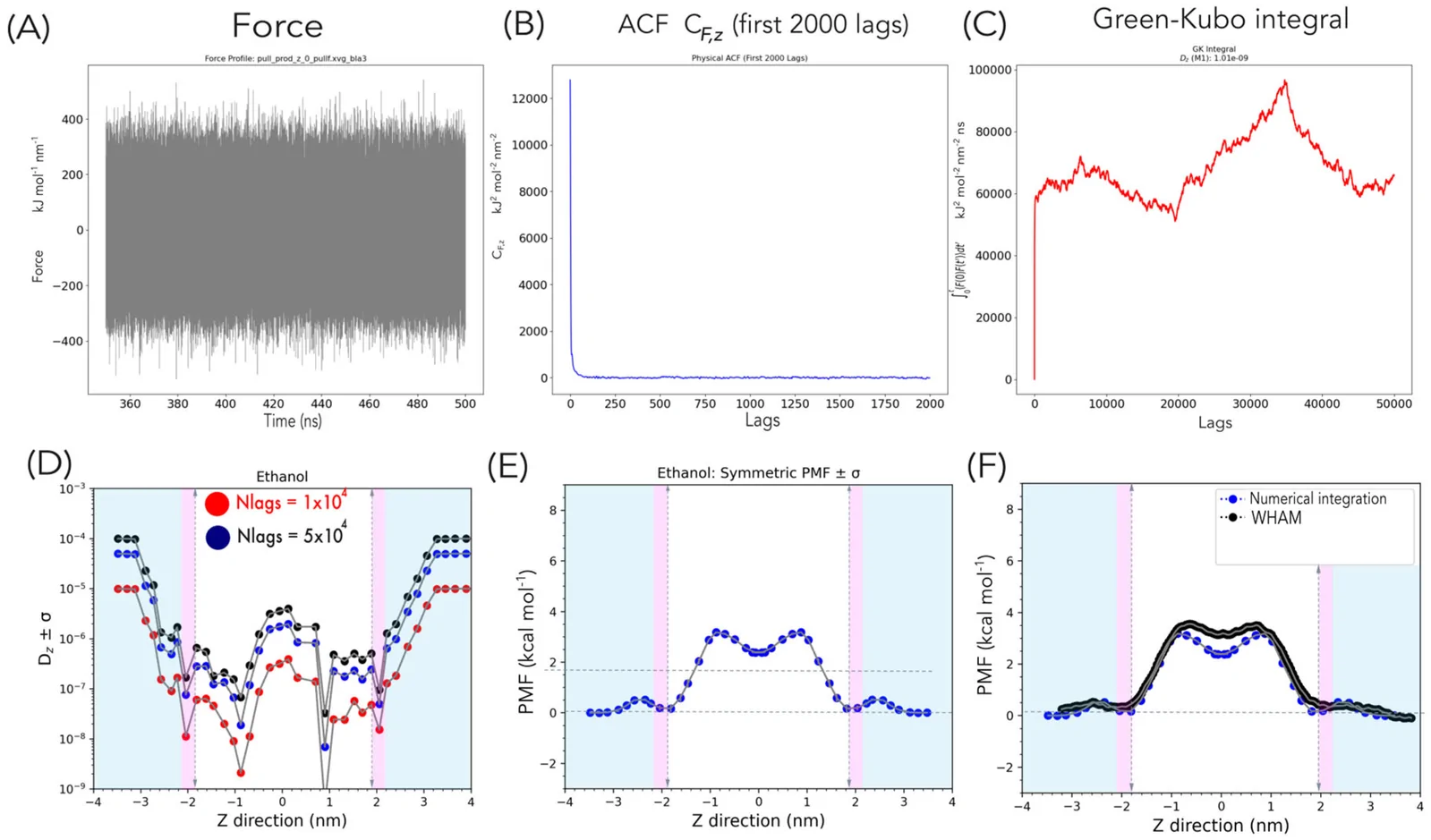
Kinetic extraction and thermodynamic profiling for simulated permeability using Constant Velocity Steered Molecular Dynamics (cv-SMD). Analysis of a 500 ns per simulation window, with (**A**–**C**) assessing 1 window, and (**D**–**F**) assessing the combination of all windows for the drug ethanol over the atomistic BBB membrane model. (**A**) The converged portion of the raw force (kJ mol^−1^ nm^−1^) time series extracted from a specific spatial window. (**B**) The unnormalised force autocorrelation function, *C_F_*_,*z*_, calculated from the raw force and shown for the first 2000 lags. (**C**) The corresponding integral of the autocorrelation function, *C_F_*, evaluated over 5 × 10^4^ lags. (**D**) Position-dependent diffusivity, *D_z_*, across the membrane coordinate. The initial 2000 lags of the autocorrelation function were fitted to a general exponential and reweighted by (*RT*)^2^. The plot demonstrates that diffusivity reaches consistency at 5 × 10^4^ lags compared to 10^4^ and 10^5^ lags. (**E**) The symmetrised Potential of Mean Force (PMF) curve obtained via numerical integration of the average force, $\int \left\langle F\left(z\right)\right\rangle \mathrm{dz}$, with symmetrisation applied at *z* = 0. (**F**) An overlay comparison of the resulting PMF curves generated via numerical integration versus those obtained using the WHAM (gmx wham). For panels (**E**,**F**), error bars represent the standard deviation (*σ*) calculated at three time points: 400 ns, 450 ns, and 500 ns. Background color coding denotes local membrane environments: blue indicates the bulk water region, pink highlights the polar headgroup interface, and the central white region represents the hydrophobic membrane core. Data and images reproduced from our previous work https://doi.org/10.1021/acs.jcim.6c01624 [85]. © 2026 American Chemical Society. Date accessed: 1 August 2026.

The decay timescale (τ_c_) tells us how “free” the molecule is to move using Brownian motion theory. Using the Green–Kubo relations [123], the autocorrelation function *C_F_* is the integral of the force autocorrelation function (as shown in Figure 7C when calculated over 5 × 10^4^ lags). The position-dependent diffusion coefficient *D*(*z*) can be calculated using the Einstein-Smoluchowski relation, it relates the thermal energy of the system to the integrated force autocorrelation function in Equation (12).

*D*(*Z*) = (*RT*)_2_/*C_F_* (12)

where *R* is the molar gas constant (≈8.314 J·mol^−1^·K^−1^) and *T* is the temperature. And $C_{F}(z)=\int_{0}^{\infty }\left\langle \Delta F(z,t)\;\Delta F(z,0)\right\rangle \;\mathrm{dt}$ is the time integral of the force autocorrelation function. Figure 7D shows this position-dependent diffusivity mapped across the different membrane environments. The plot shows that the calculated diffusivity reaches stable consistency when integrated over 5 × 10^4^ lags, as opposed to shorter (1 × 10^4^) or longer (1 × 10^5^) intervals. The force autocorrelation function, *C_F,t_* can be computed from these fluctuations from *t*_0_ to infinity to quantify the memory effects of the local environment. However, a lot of these profiles do not converge at long timescales, and as such, approximation methods are relied on [85]. This function indicates how much the force at one point in time (as shown in Figure 7A when using 350 to 500 ns) influences the force at a subsequent time (known as lag time, *t*) [124]. We direct readers to our recent publication [85], which explores the reasons for the underlying parameters, binning, lag-time selection, convergence, and model validation. The force autocorrelation function can be computed to quantify memory effects of the local environment. A rapid decay of *C_F,t_* indicates low friction and enhanced mobility, whereas slower decay corresponds to increased resistance.

The transcellular permeability can then be calculated by integrating position-dependent profiles obtained from US simulations: namely the position-dependent diffusion *D*(*z*) and free energy ∆*G*(*z*) along the transition coordinate *Z*, as shown in the ISD equation (Equation (4)). The symmetrised Potential of Mean Force (PMF) curve is derived via numerical integration of the average force, as depicted in Figure 7E. An overlay comparison (Figure 7F) demonstrates strong alignment between the PMF generated via direct numerical integration and the profile reconstructed using WHAM.

### 4.8. Comparison of Different Enhanced Sampling Methods

In Table 2, we provide a summary of different enhanced sampling methods, their biasing mechanism, key strengths, and core limitations. We now proceed to comment on some of these. While Umbrella Sampling (US) is widely used for assessing small-molecule permeation, its accuracy relies heavily on CV quality and spatial window separation [109]. This means diffusivity can be explored across the system without predefined windows. When validated against experimental metrics, ABF frequently achieves statistical convergence comparable to or superior to US, provided orthogonal degrees of freedom are adequately captured [121]. Metadynamics addresses the limitations of rigid reaction coordinates by saving history-dependent bias potentials along selected CVs to discourage the system from revisiting sampled conformational states [118]. Variants such as Well-Tempered (WT-MetaD) and Multiple-Walker Metadynamics (MW-MetaD) further enhance efficiency, where MW-MetaD accelerates convergence by parallelising the exploration of the free-energy surface across multiple replicas that share a common bias history.

Despite these advantages, these methods share a fundamental vulnerability: when orthogonal relaxation times exceed the simulation window, hidden energy barriers perpendicular to the chosen CV induce quasi-non-ergodicity, leading to hysteresis and systematic discrepancies compared to experimental thermodynamic limits [94,121]. Consequently, sampling protocols must pair enhanced sampling with corrective kinetic frameworks. To address these limitations within the hBMEC model, this review highlights a sequential method using cv-SMD to generate initial structural configurations, followed by US combined with WHAM, DHAM, and the ISD framework [85,104]. By employing a transition-based Markov State Model framework to evaluate observed transitions between spatial bins, DHAM mitigates artifacts from incomplete sampling and transient trapping that frequently affect simulations with complex membranes [43,127]. The ISD framework is applied to US windows, allowing for the derivation of the *P_sim_* of a drug using the thermodynamic and local diffusivity data [85].

One important aspect of the proliferation of enhanced sampling methods is that they be carefully evaluated against experimental benchmark data. When this benchmarking is done, it becomes apparent that users need to assess computational convergence, collective variable (CV) setups, and the inherent statistical precision of their method of choice. Umbrella Sampling (US) remains a central technique for assessing small-molecule membrane permeation. However, its accuracy against experimental observables, including NMR scalar couplings [128], FRET distances, or permeabilities, is heavily determined by the quality of the CV and the window separation [129,130]. In contrast, Adaptive Biasing Force (ABF) [131] continuously estimates and cancels the thermodynamic force along the CV, allowing diffusive exploration of the free energy surface without requiring prior knowledge of the underlying potential profile. When validated against experimental metrics like hydration free energies or single-molecule mechanical unfolding pathways, ABF frequently achieves comparable or superior statistical convergence to US, particularly when orthogonal degrees of freedom are captured [121].

In quantitative comparisons targeting experimental observables (e.g., small-molecule membrane transfer free energies and protein folding landscapes), Metadynamics often demonstrates higher computational efficiency and lower statistical uncertainty than standard US for equivalent sampling times [119,132]. However, all three methods share a fundamental vulnerability: when orthogonal relaxation times exceed the simulation window, hidden energy barriers perpendicular to the chosen CV induce quasi-non-ergodicity, leading to hysteresis and systematic discrepancies when compared to experimental thermodynamic limits. Consequently, state-of-the-art sampling protocols pair enhanced sampling methods with corrective procedures or experimental constraints to address these limits.

## 5. Conclusions

In this work, we covered the basics of the blood–brain barrier (BBB) endothelial membrane, a highly heterogeneous membrane whose composition limits the transport into the brain to just 2% of the 10^60^ molecular drug space. We covered the details of the human brain microvascular endothelial cells (hBMECs).

We reviewed a number of computational techniques that can be used to assess BBB transport and CNS delivery, starting with molecular dynamics (MD) simulations for predicting in silico drug permeability across an atomistic hBMEC model. We highlight the Inhomogeneous Solubility–Diffusion (ISD) as the primary framework linking the transmembrane permeability, *P*_sim_, to the Gibbs Free Energy of permeation and the z-diffusivity of permeation. Due to the very slow timescales to sample CNS transport a priori with MD simulations, we compared MD with transition-based counting (TBC) methodology to several enhanced sampling techniques. The latter techniques are a solution to this problem of reduced or insufficient sampling with unbiased MD simulations. We covered the following enhanced sampling simulations: (i) umbrella sampling (US) with the Weighted Histogram Analysis Method (WHAM) and the Dynamic Histogram Analysis Method (DHAM), and (ii) constant velocity-steered molecular dynamics (cv-SMD)/US combined with the ISD model, as well as commenting on Metadynamics and the Adaptive Biasing Force (ABF) methods.

## Figures and Tables

**Table 1:** Composition of BBB apical endothelial membrane. The general principles of the endothelial membranes are laid out in previous work [91]. Some governing principles include the need for ~30% cholesterol, sphingomyelin lipids at ~20%, and the remainder consisting of phospholipids. N-oleoyl-sphingomyelin (OSM; 18), 1-palmitoyl-2-oleoyl-glycero-3-phosphocholine (POPC; 4), 1-stearoyl-2-arachidonoyl-sn-glycero-3-phosphocholine (SAPC; 8), 1-stearoyl-2 arachidonoyl-sn-glycero-3-phosphoethanolamine (SAPE; 14), 1-stearoyl-2-oleoyl-sn-glycero-3-phosphoethanolamine (SOPE; 6), 1-stearoyl-2-arachidonoyl-sn-glycero-3-phospho-L-serine (SAPS; 8), 1-stearoyl-2-linoleoyl-sn-glycero-3-phosphocholine (SLPC; 8), 1-stearoyl-2-arachidonoyl-sn-glycero-3-phosphoinositol (SAPI; 2) and cholesterol (28).

Lipid	Number	% Composition	Justification
Cholesterol	28	29.1	Cholesterol component
OSM	18	18.8	Sphingomyelin component
SAPE	14	14.5	Phospholipid component
SAPC	8	8.3	Phospholipid component
SAPS	8	8.3	Phospholipid component
SLPC	8	8.3	Phospholipid component
SOPE	6	6.3	Phospholipid component
POPC	4	4.1	Phospholipid component
SAPI	2	2.0	Phospholipid component
Total	96	100	-

**Table 2:** Comparison of enhanced sampling techniques for membrane permeation studies. We compare unbiased MD with transition-based counting, Adaptive Biasing Force (ABF), high-temperature unbiased MD, metadynamics, US with WHAM/DHAM, and cv-SMD/US with ISD [125].

Technique	Biasing Mechanism	Key Strengths	Core Limitations
Unbiased MD with Transition-Based Counting [22,40]	None—drug moves freely under relevant conditions.	Mimics real-world conditions.	Complex systems may require unreasonable computational timeframes to observe rare events and achieve convergence. Therefore, this method remains intractable for all but the fastest molecules.
Adaptive Biasing Force (ABF) [94,121]	Computes the local mean force along a reaction coordinate and applies equal and opposite biasing force to dynamically flatten the free energy landscape.	Does not need predefined windows or post-simulation unbiasing techniques.	Complex systems possess many slow and coupled degrees of freedom, making convergence difficult to achieve in a reasonable timeframe.
High-Temperature Unbiased MD [52,91,126]	High temperatures enhance the kinetic energy available to the system.	Increases rare events such as spontaneous membrane permeation without the need for unbiasing.	Relies on unrepresentative temperatures, often using parameters that were not created for the temperature used; results require extrapolation for lower temperatures.
Metadynamics [118]	Uses history-dependent bias potentials along selected collective variables to discourage the system from revisiting previously sampled conformational states.	Effective at displacing systems from deep local minima; is capable of simultaneously biasing multiple collective variables.	Requires precise parameterisation of bias height, width, and deposition rate to prevent the over-filling of free energy basins.
US with WHAM/DHAM [43,104,127]	Uses static harmonic restraining potentials to enforce sampling within specific, overlapping spatial windows along a defined reaction coordinate.	Facilitates high parallelisation; the integration of WHAM allows for free energy extraction, and DHAM enables the evaluation of observed transitions between spatial bins to derive kinetic transition rates.	Sensitive to initial configurations and slow local relaxation times within individual windows; requires verification of inter-window overlap; requires computationally expensive simulation times to achieve convergence.
cv-SMD/US with ISD [85]	Applies a virtual harmonic spring to pull a molecule along a reaction coordinate at a constant velocity, directly measuring the required force.	Allows the direct integration of force to identify the energetic landscape.	Careful selection of pulling velocity is needed to avoid non-equilibrium work; requires computationally expensive simulation times to achieve convergence.

## Data Availability

No new data were created or analyzed in this study. Data sharing is not applicable to this article.

## Abbreviations

The following abbreviations are used in this manuscript:

ABC	ATP-Binding Cassette
AFM	Atomic Force Microscopy
AMBER	Assisted Model Building with Energy Refinement
BBB	Blood–Brain Barrier
BCSFB	Blood–Cerebrospinal Fluid Barrier
BM	Basement Membrane
cf-SMD	Constant Force-Steered Molecular Dynamics
CG	Coarse-Graining
CHARMM	Chemistry at Harvard Macromolecular Mechanics
CNS	Central Nervous System
cv-SMD	Constant Velocity-Steered Molecular Dynamics
DPPC	1,2-dihexadecanoyl-sn-glycero-3-phosphocholine
DHAM	Dynamic Histogram Analysis Method
DPPG	1,2-Dipalmitoyl-sn-glycero-3-phosphoglycerol
ER	Endoplasmic Reticulum
ERAD	Endoplasmic Reticulum-Associated Degradation
FDA	Food and Drug Administration
FF	Force Field
GROMOS	Groningen Molecular Simulation
hBMEC	Human Brain Microvascular Endothelial Cell
ISD	Inhomogeneous Solubility–Diffusion
ISF	Interstitial Fluid
MD	Molecular Dynamics
MM	Molecular Mechanics
MMFF	Merck Molecular Force Field
MSM	Markov State Model/Modelling
MW	Molecular Weight
NPT	Isothermal-Isobaric Ensemble (Constant number of particles, pressure, and temperature)
NVT	Canonical Ensemble (Constant number of particles, volume, and temperature)
OPLS	Optimised Potentials for Liquid Simulations
OSM	N-oleoyl-sphingomyelin
P-gp	P-glycoprotein
PMF	Potential of Mean Force
PNS	Peripheral Nervous Systems
POPC	1-palmitoyl-2-oleoyl-glycero-3-phosphocholine
QM	Quantum Mechanics
QSAR	Quantitative Structure–Activity Relationship
SAPC	1-stearoyl-2-arachidonoyl-sn-glycero-3-phosphocholine
SAPE	1-stearoyl-2-arachidonoyl-sn-glycero-3-phosphoethanolamine
SAPI	1-stearoyl-2-arachidonoyl-sn-glycero-3-phosphoinositol
SAPS	1-stearoyl-2-arachidonoyl-sn-glycero-3-phospho-L-serine
SLPC	1-stearoyl-2-linoleoyl-sn-glycero-3-phosphocholine
SMD	Steered Molecular Dynamics
SOPE	1-stearoyl-2-oleoyl-sn-glycero-3-phosphoethanolamine
WHAM	Weighted Histogram Analysis Method
